# Expression of transferrin receptor‐1 (TFR‐1) in canine osteosarcomas

**DOI:** 10.1002/vms3.258

**Published:** 2020-04-02

**Authors:** Gionata De Vico, Manuela Martano, Paola Maiolino, Francesca Carella, Leonardo Leonardi

**Affiliations:** ^1^ Department of Biology University of Naples Federico II Complesso universitario di Monte S. Angelo Naples Italy; ^2^ Department of Veterinary Medicine and Animal Productions University of Naples Federico II Naples Italy; ^3^ Department of Veterinary Medicine University of Perugia Perugia Italy

**Keywords:** Canine Osteosarcoma, immunohistochemistry, Transferrin Receptor‐1

## Abstract

Due to high rates of proliferation and DNA synthesis, neoplastic cells have higher requirements of iron than normal cells. For that reason, neoplastic cells have remodelled iron metabolism pathways, over‐expressing genes encoding for iron uptake proteins, among which Transferrin Receptor‐1 (TFR‐1). Accumulating evidence has proven that overexpression of TFR‐1 and high Iron concentration, are both widespread condition of cancer cells, both essential to tumour onset and progression. We studied TFR‐1 and PCNA immunohistochemical expression in fifteen (15) Canine osteoblastic osteosarcomas (COS). After immunohistochemical staining, counting of TFR‐1 positive cells by two independent observers showed that 85%–95% of neoplastic cells were strongly labelled at cytoplasmic level by anti‐TFR‐1 antibody in all examined COS. Furthermore, 70%–80% of neoplastic cells were positively labelled at the nuclear level by PCNA. Surprisingly, about 100% of intratumour vascular endothelial cells were also positive, whereas extratumour vascular endothelial cells were negative. The latter is an interesting finding, as TFR‐1 is usually not expressed in normal vasculature, with the exception of normal brain vascular endothelium, where it allows transport of transferrin, and thus iron, into tissues, suggesting a similar function here to support cancer growth. The early results presented highlight the relevance of TFR‐1 expression in canine OS, suggesting therapies involving both TFR‐1 and Iron metabolisms in dogs with osteosarcoma should be developed.

## Graphical abstract

Neoplastic cells have higher requirements of iron than normal cells. For that reason, neoplastic cells over‐express genes encoding for iron uptake proteins, including Transferrin Receptor‐1 (TFR‐1). We studied TFR‐1 immunohistochemical expression in ten Canine osteoblastic osteosarcomas (OS). In all examined OS, 85–95% of neoplastic cells showed a strong cytoplasmic immunostaining with anti‐TFR‐1 antibody. Surprisingly, about 100% of intratumor vascular endothelial cells were also positive, whilst extra‐tumor vascular endothelial cells were negative. The early results presented highlight the relevance of TFR‐1 expression in canine OS, suggesting therapies involving both TFR‐1 and Iron metabolisms in dogs with osteosarcoma should be developed.

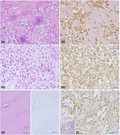

## 1 INTRODUCTION

Iron is known to play a crucial role in tumour cells metabolism, proliferation and growth (Kazan, Urfali‐Mamatoglu, & Gunduz, 2017)**.** Due to high rates of proliferation and DNA synthesis, neoplastic cells have higher requirements of iron than normal cells, a phenomenon termed as ‘iron addiction’ (Manz, Blanchette, Paul, Torti, & Torti, 2016). To satisfy the high requirement of iron, neoplastic cells have remodelled iron metabolism pathways, over‐expressing genes encoding for iron uptake proteins (Kazan et al., 2017; Shen et al., 2018). Among these, transferrin receptor‐1 (TFR‐1 or CD71), a member of the TFR family, is able to form complexes with transferrin (TF) bound to Fe (III), whose internalization represents the most important way for cancer cells to absorb iron (Daniels, Delgado, Helguera, Penichet, & Tracy, 2006; Daniels, Delgado, Rodriguez, Helguera, & Penichet, 2006; Shen et al., 2018). Accumulating evidence has proven that overexpression of TFR‐1 and high iron concentration, are both widespread conditions of cancer cells, and both seem to be essential to tumour onset and progression (Daniels, Delgado, Helguera, et al., 2006; Daniels, Delgado, Rodriguez, et al., 2006; Shen et al., 2018), and can represent potential selective targets in cancer therapy (Daniels, Delgado, Helguera, et al., 2006; Daniels, Delgado, Rodriguez, et al., 2006; Shen et al., 2018).

Dysregulated expression of TFR‐1 has been reported in many human tumours (Shen et al., 2018). Unfortunately, studies of TFR‐1 expression in canine tumours is limited to canine brain tumour (Olby, Munana, Sharp, Skeen, & Hauck, 2000), canine lymphomas (Priest, McDonough, Erb, Daddona, & Stokol, 2011) and canine malignant oronasal tumours (Ployptech et al., 2017)**.** Osteosarcoma (OS) is the most common primary bone tumour in dogs, tends to occur in middle‐aged to older dogs (median age of 7 years), in large and giant breeds more frequently at the metaphyseal region of long bones (Mueller, Fuchs, & Kaser‐Hotz, 2007; Rankin et al., 2012). Canine osteosarcoma (COS), is a well recognized model for human osteosarcoma (Mueller et al., 2007; Rankin et al., 2012). In this report, we provide evidence for the increased expression of TFR‐1 in COS, suggesting both Iron oxidative metabolism and TFR‐1 could be useful potential new targets in the management of the disease.

**Figure 1:**
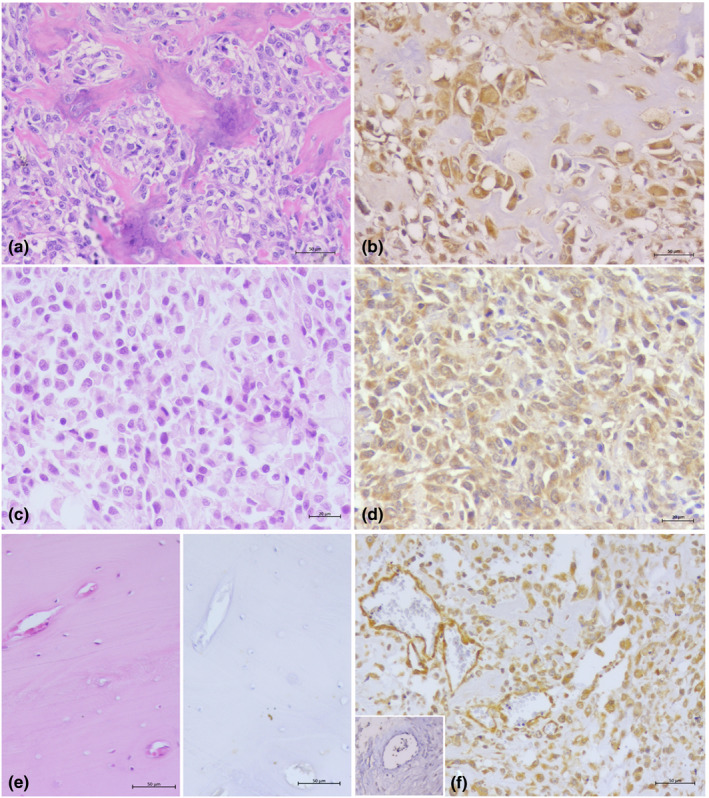
Canine Osteosarcomas (COS) – (a) Productive type, Haematoxylin and Eosin (HE); (b) Productive type TFR‐1 Immunestaining (note the strong cytoplasmic staining of neoplastic cells); (c) Non‐Productive type (HE); (d) Non‐Productive type, TFR‐1 Immunestaining (about 90% of cells are TFR‐1 positive); (e) Normal bone HE(Left) and TFR‐1 Immunestaining (Rigth, negative); (f) OS Intratumour vasculature (Large figure, endothelial vasculature is TFR‐1 positive); Exrtratumour vessels are TFR‐1 negative (Insert)

Fifteen (15) samples of COS were taken from dogs of different breeds, sex and age (see Table 1). Samples were 10% formalin fixed, paraffin embedded for routine histological processing and stained with haematoxylin and eosin for light microscopy study. Tumours were classified according to the World Health Organization criteria as osteoblastic type productive (n. 8) and non‐productive (n.7)(Slayter et al., 1994). Additional sections from each COS were treated for TFR‐1 immunohistochemistry and for PCNA immunohistochemistry, respectively. Sections from normal bone were also tested as negative control. Briefly, after deparaffinization in xylene, slides were rehydrated through graded ethanols and rinsed in distilled water. Endogenous peroxidase activity was blocked with a 3% solution of hydrogen peroxide in methanol for 10 min. Following a rinse in distilled water, slides were heated by microwave (high, 800 W) for 20 min in boiling EDTA buffer (pH 8.0) for antigen retrieval. Slides were then washed with distilled water and phosphate‐buffered saline (PBS; pH 7.4) before blocking with normal goat serum (Vector Laboratories) and a casein solution (Vector Laboratories). Immunohistochemical staining for TFR‐1 was then performed with a mouse antihuman TFR‐1 antibody (clone H68.4, Zymed Laboratories, San Francisco, CA), diluted to 1:50 in a solution of casein and PBS, for a period of 90 min (Priest et al., 2011). According to Maiolino, Restucci, and Vico (1995) proliferating cell nuclear antigen (PCNA) immunohistochemistry, was performed using a mouse monoclonal antibody against PCNA, clone PC10 (Sigma) incubated overnight at 4°C with the primary antibody (dilution 1:300). The number of positively labelled cells for TFR‐1 and for PCNA, respectively, was examined in each specimen by two independent observers (GDV and MP) under blinded conditions, by counting 1.000 cells in 10 fields at 400X magnification (40X objective 10X ocular) and results were expressed as percentage. Independently of the tumour type, 85%–95% of neoplastic cells showed a strong cytoplasmic immunostaining for TFR‐1 antibody in all examined COS. Furthermore, 70%–80% of neoplastic cells were positively labelled at the nuclear level by PCNA*.* Surprisingly, about 100% of intratumour vascular endothelial cells were also positive, whereas extratumour vascular endothelial cells were negative (Figure 1).. Previous reports in canine lymphomas, showed that in 95% of cases examined by Priest et al. (2011), more than 75% of the neoplastic cells expressed TFR‐1; in canine brain tumours, TFR‐1 expression was stronger in primary and metastatic tumours than in normal brain tissue (Olby et al., 2000). Finally, TFR‐1 expression in canine oronasal cancer correlated well with tumour stage (Ploypetch et a., 2017). An interesting finding of our work concerns the strong positivity of intratumour vascular endothelial cells to TFR‐1. Little is known about the regulatory mechanisms that influence the selective expression of the TFR gene in endothelial vascular cells (Holloway, Sade, Romero, & Male, 2007). TFR is usually not expressed on vascular endothelium, whereas it is normally expressed in human brain vascular endothelium, where it allows transport of transferrin and thus iron into tissues (Fishman, Rubin, Handrahan, Connor, & Fine, 1987). This finding could suggest a similar function in cancer cells supporting their growth. Furthermore, the high PCNA index observed in our cases, support the opinion that TFR expression could be related to the proliferative activity of neoplastic cells, which is related to the malignant behaviour in canine osteosarcomas (Dolkaet, Sapierzyński, & Król, 2013). The control of TFR‐1 expression in different cell types appears to occur at the transcriptional level (Holloway et al., 2007). However, the expression of the TFR may also be controlled by a post‐translational mechanism in response to iron demand (Holloway et al., 2007). Further studies could better elucidate the mechanism involved in the above regulation in normal and neoplastic tissues. The widespread overexpression of TFR‐1 in such a great number and type of tumours, highlight its relevance as a potential target for therapy (Shen at al., 2018). Studies suggest that TFR‐1 could be targeted in order to deliver therapeutic agents into tumour cells by receptor‐mediated endocytosis, or to inhibit cell growth and/or induce apoptosis in malignant cells using cytotoxic anti‐TFR‐1 antibodies (Daniels, Delgado, Helguera, et al., 2006; Daniels, Delgado, Rodriguez, et al., 2006). Furthermore, tumours expressing TFR‐1, could be also targeted by drugs able to modulate intracellular iron oxidative state (Daniels, Delgado, Helguera, et al., 2006; Daniels, Delgado, Rodriguez, et al., 2006), in order to induce ferroptosis in malignant cells. Ferroptosis is a regulated form of non‐apoptotic cell death driven by accumulation of lipid‐based reactive oxygen species (ROS), particularly lipid hydroperoxides, in the presence of catalytically active iron (Yang & Stockwell, 2016). It has become evident that ferroptosis is a druggable pathway with tractable nodes that can be modulated by several drugs (Seibt, Proneth, & Conrad, 2019). Among these, erastin inhibits cystine uptake by the cystine/glutamate antiporter, creating a void in the antioxidant defences of the cell (Dixon et al., 2012). The anticancer activity of artemisinin, a sesquiterpene lactone which is the mine phytochemical component of the plant *Artemisia annua* (Zarrelli, Pollio, & Aceto, 2019), is also associated with the presence of iron (Ookoa et al., 2015). This metal, present in large excess in cancer cells, fosters the cleavage of artemisinin's endoperoxide bridge in a Fenton‐type chemical reaction. This leads to the generation of ROS,which induce cell death by ferroptosis (Ookoa et al., 2015). Interestingly, both human OS and COS cell lines are sensitive to dihidroartemisinin, a metabolic derivative of artemisinin (Hosoya et al., 2008; Jirangkul, Srisawat, Punyaratabandhu, Songpattanaslip, & Mungthin, 2014). Although in normal tissues TFR is found in a limited number of sites, these include, however, some belonging to the haematopoietic lineages (Lesley at al., 1984), thus bone marrow toxicity must always be carefully monitored when considering use TFR as target for therapy. Early studies, suggest that anti‐TFR1 immunotoxins may have efficacy when administered locally in certain cases, but toxicity is a concern in the case of systemic administration; however, the problem may be overcome by changing the delivered cargo, such as using immunoliposomes (Daniels‐Wells & Penichet, 2016). Furthermore, both in vitro and in vivo studies showed a vast types of anticancer drugs have been delivered into cancer cells employing a variety of TFR‐binding molecules (such as TF, anti‐TFR antibodies, or TFR‐binding peptides) (Daniels‐wells et al., 2012). The delivery of therapeutic molecules directly into malignant cells results in increasing intracellular drug concentration and in more effective tumour targeting, with a greatly reduced general toxicity, bone marrow toxicity and myelosuppression, and therefore in an overall increased therapeutic efficacy (Daniels et al., 2012).

**Table 1:** Breeds, sex, age and osteosarcoma (OS) types studied

Breed	Sex	Age (ys)	OS type
Mixed Breed	M	13	Productive
Dobermann	M	6	Productive
Mixed Breed	Mn	12	Non‐productive
Mixed Breed	F	nd	Productive
Mixed Breed	F	11	Non‐productive
Rottweiler	M	10	Non‐productive
Rottweiler	nd	nd	Productive
Rottweiler	nd	nd	Productive
Terranova	F	8	Productive
Terranova	F	8	Non‐productive
Mixed Breed	M	9	Productive
German Shepherd	F	7	Productive
German Shepherd	M	8	Non‐productive
Boxer	F	7	Non‐productive
Dobermann	M	7	Non‐productive

Abbreviations: *F*, Female; *M*, Male; Mn, neutered Male; nd, not determined.

Regarding toxicity of artemisinin and its derivatives we have now early results in dogs which suggest that COS cell lines are sensitive to dihidroartemisinin, a metabolic derivative of artemisinin (Hosoya et al., 2008; Jirangkul et al., 2014). Furthermore, in vivo studies support the opinion that these drugs are safe and effective in treating canine cancer, with minor and transient toxic effects (Breuer & Efferth, 2014; Rutteman et al., 2013). Further studies are needed to investigate the underlying molecular basis of the high TFR‐1 expression in COS. Furthermore, the comparative study of this molecule in human OS is also strongly suggested. However, the early results presented in this study, though on a limited number of cases, highlight the relevance of TFR‐1 expression in COS, suggesting therapies involving both TFR‐1 and iron metabolisms in dogs with osteosarcoma should be developed.

## CONFLICT OF INTERESTS

The authors declare no conflict of interests.

## AUTHOR CONTRIBUTION

**Gionata De Vico**: Conceptualization; Data curation; Formal analysis; Investigation; Methodology; Writing‐original draft; Writing‐review & editing. **Manuela Martano**: Formal analysis; Investigation; Validation. **Paola Maiolino**: Data curation; Formal analysis; Methodology; Validation. **Francesca Carella**: Data curation; Formal analysis; Investigation. **Leonardo Leonardi**: Data curation; Formal analysis; Investigation; Methodology.

## ETHICAL STATEMENT

The authors confirm that the ethical policies of the journal, as noted on the journal's author guidelines page, have been adhered to. No ethical approval was required as all samples were from routine diagnostic archival material.
